# Using Dry Needling to Improve the Quality of Life of Patients With Shoulder Dysfunction Following Neck Dissection: An Innovative Case Report

**DOI:** 10.7759/cureus.58541

**Published:** 2024-04-18

**Authors:** Kalyani P, Mahathi Neralla, Senthilnathan P, Dharmesh Kubendiran, Ravalika Singarapu

**Affiliations:** 1 Department of Oral and Maxillofacial Surgery, Saveetha Dental College and Hospitals, Saveetha Institute of Technical and Medical Sciences, Saveetha University, Chennai, IND

**Keywords:** oral cancer, case series, neck dissection, shoulder disability, dry needling

## Abstract

The spinal accessory nerve manipulation or sacrifice during neck dissection results in trapezius muscle denervation and atrophy, leading to shoulder disability. Patients start experiencing pain and weakness while moving their shoulders, including elevation, rotation, and abduction, as well as reduced range of motion (ROM) and dropping of the shoulders. There are several ways to treat the condition, including using painkillers or undergoing physical therapy. Physical therapy plays a major role in improving shoulder function. Dry needling (DN) is an emerging treatment modality that involves eliciting a local twitch response in the region of myofascial trigger points, which can reduce pain and increase the ROM. This case report documents how DN improved shoulder function in a 51-year-old female who had pain when moving the shoulders and limited ROM after undergoing a modified radical neck dissection.

## Introduction

Quality of life (QoL) is defined as an individual’s perception of their position in life in the context of the culture and value systems in which they live and in relation to their goals, expectations, standards, and concerns. In other words, it is the perceived discrepancy between the actual condition and the ideal standards of the patient [1]. Surgical treatment for cancers affecting oral and maxillofacial regions has a significant impact on a patient’s physical, social, functional, and psychological well-being, which can, in turn, affect the QoL negatively. In recent years, the QoL of a patient who received treatment for any disease has become a significant factor in evaluating the prognosis of the therapeutic procedure [2]. Shoulder dysfunction after neck dissection and spinal accessory nerve manipulation can negatively affect postsurgical QoL if not properly rehabilitated. Myofascial trigger points (MTrPs) in the shoulder muscles are common in patients with shoulder pain. Muscle stretching, compression, or contraction may lead to pain in the presence of MTrPs [2]. These MTrPs are hyperirritable points in taut bands of the skeletal muscle and are painful on compression, producing motor dysfunction, referred pain, and restricted movements due to stiffness of the muscle. The most common muscle affected by the MTrPs is the upper trapezius, with symptoms including taut and painful muscle, tension headache, and neck pain [3,4]. Uncommonly, patients with these conditions may also experience dizziness, vertigo, and limitation in the range of motion (ROM) of the neck and shoulder joint [3,4]. The upper trapezius muscle is involved in the scapulohumeral rhythm during shoulder movement, and hence, trigger points in these muscles can lead to shoulder dysfunction and instability [5,6].

There are also various conservative treatment modalities available for the release of these MTrPs, such as wet needling (e.g., lidocaine injection and some local anesthetic injections), laser therapy, and ischemic compression apart from dry needling (DN) and oral drug administration [7]. DN is simple and effective and is currently being used widely. DN inactivates the MTrPs, normalizes the chemical environment of MTrPs, releases muscle shortening by removing the cause, normalizes the peripheral nerve sensitization by promoting self-healing of the injured tissue, and, most importantly, reduces spontaneous muscle activity. DN stimulates the A-delta nerve fibers, activating the enkephalinergic inhibitory dorsal horn interneurons, leading to pain relief, which is opioid-mediated. The local twitch response after DN can also correct the increased levels of bradykinin, calcitonin gene-related peptide, substance P, and other chemicals at MTrPs by increasing the microcirculation and blood flow in the muscle [8].

## Case presentation

**Figure 1:**
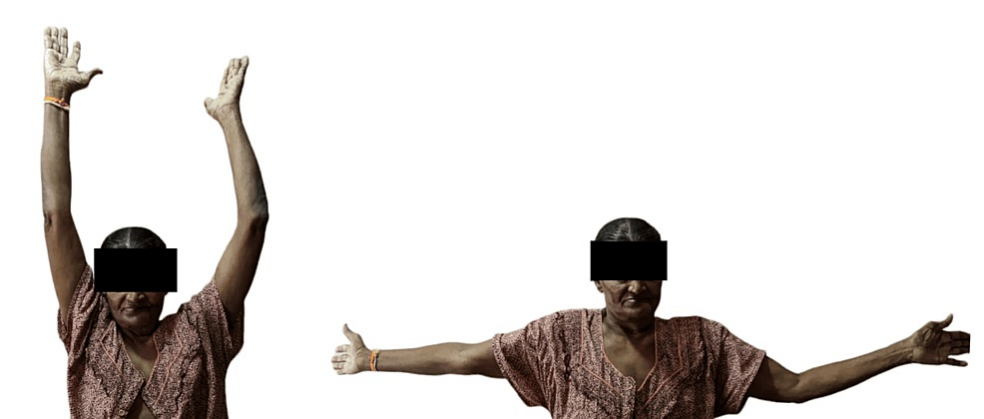
Pretreatment picture displaying weakness in the left shoulder

The patient was a 51-year-old female who was diagnosed with squamous cell carcinoma on the left lateral border of the tongue. Surgery included left partial glossectomy and left modified radical neck dissection (MRND) while preserving the spinal accessory nerve, sternocleidomastoid, and internal jugular vein. The tongue was closed primarily. The main postoperative complaints were pain in the left upper scapular region and limited range of movement of the left shoulder. She mentioned that she had been experiencing these symptoms since her surgery. Her main concern was weakness in her left shoulder (Figure 1).

Upon examination, she had trapezius muscle atrophy, shoulder droop, and depressed and protracted scapula on the operated side. Signs of trapezius muscle palsy and spinal accessory nerve dysfunction were evident on the operated side. Upon assessment, her Visual Analog Scale (VAS) score was found to be 8, and she was found to have limited ROM.

**Figure 2:**
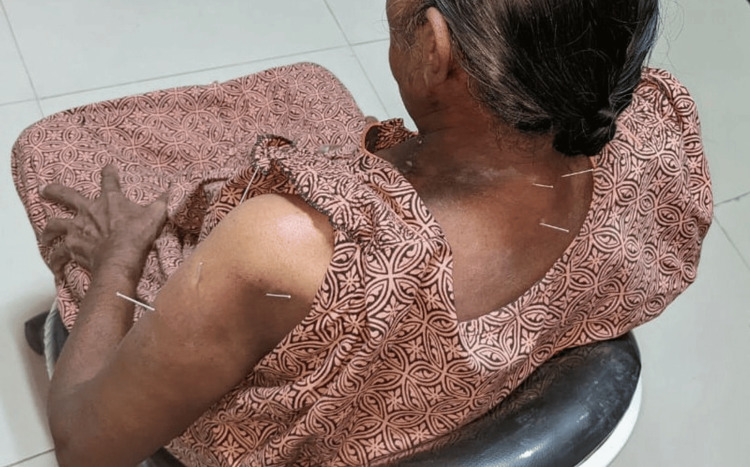
Dry needling of the trigger points in the trapezius and the deltoid muscles

The purpose of DN intervention was to maintain the passive ROM of the glenohumeral joint, increase muscular strength, and decrease the associated pain. The DN technique was used on the following muscles: supraspinatus, infraspinatus, paraspinal muscles from C6-T2, supraspinatus tendon, infraspinatus tendon, deltoid muscle (anterior, posterior, and middle), deltoid tendon, and trapezius muscle (superior and inferior) (Figure 2). Shoulder exercises targeting flexion and ROM consisted of simple wall stretches using single arm and double arms. Shoulder abduction and shoulder rotation (forward and backward) were also taught. Exercises such as standard trapezius stretches, shoulder shrugs, and isometrics focused on improving strength. Flexion and abduction isometric exercises used the patient's other hand as resistance. Each exercise was instructed to be performed in three sets of 25 repetitions three times a day.

**Figure 3:**
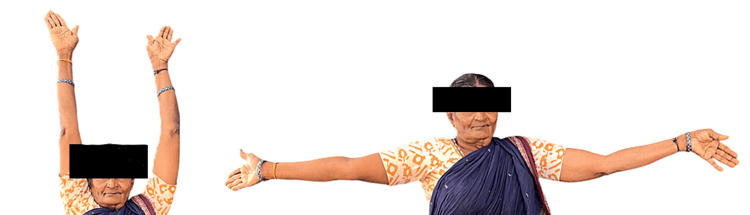
Posttreatment picture showing improvement in the left shoulder

These sessions were conducted on a weekly basis, with two sessions per week, for a period of four to six weeks. Shoulder exercises were started simultaneously, and the patient was instructed to continue with them without a break. At the end of the therapy, her pain score had markedly reduced from 8 to 2. There was also a visible improvement in the ROM of her joint, as shown in Figure 3.

## Discussion

MTrPs are defined as hyperirritable spots in a palpable taut band of skeletal muscle fibers, giving rise to characteristic referred pain, motor dysfunction, and autonomic phenomena [9,10]. Trauma, overuse of the muscle, postural habits, emotional and psychological stresses, and endocrine, metabolic, and nutritional deficiencies can precipitate MTrPs [11]. In the study by Brennan et al. [12], positive changes in clinical outcomes for myofascial pain of the upper trapezius muscle were observed when only DN was used as a treatment modality, but only for the first three weeks. Hence, the authors recommended the use of DN along with intramuscular electrical stimulation to achieve better clinical outcomes.

Patients who undergo pectoralis major myocutaneous flap reconstruction experience reduced ROM in horizontal adduction, with the patient not being able to complete a full 180° adduction [13,14]. Isometric contraction of the shoulder can also be affected by these flaps. However, medial rotation and shoulder flexion are only mildly affected. Shoulder morbidity because of the pectoralis major myocutaneous flap is hard to investigate because almost all patients with a pectoralis major myocutaneous flap reconstruction undergo a neck dissection, which is known to have shoulder morbidity, especially in terms of abduction and flexion. Shoulder dysfunction is more significant in patients who undergo pectoralis major flap reconstruction with MRND than in patients who undergo MRND alone [15]. According to Para-García et al. [16], a combination of DN and physical exercise therapy can be effective in treating patients with subacromial pain. This approach resulted in a significant decrease in pain. However, it did not improve the disability index of patients with subacromial pain syndrome. It is important to consider the sequencing of different treatment modalities in treating shoulder pain. According to the guidelines of the Dutch College of General Practitioners, the initial treatment for shoulder pain for the first two weeks shall be patient education combined with the use of analgesics and nonsteroidal anti-inflammatory drugs. If there is no improvement within the first two weeks, intra-articular steroid injections can be administered and repeated for the following two weeks. Finally, after six weeks, if functional limitations are still observed, then physical therapy is advised [17]. The mechanism behind DN inhibiting pain was explained in the study by Ziaeifar et al. [18]. According to Ziaeifar et al. [18], DN elicits a local twitch response that reduces the concentration of sensitizing substances in the regions of MTrPs. Furthermore, prolonged permanent pain relief is due to the focal injury and microtrauma following needle insertion, which can last for several days. Repeated DN and microtrauma lead to the formation of scar tissue that consecutively reduces the number of functioning nociceptors, leading to pain control [19]. This intervention is a continuation of our previous study where we did a subjective assessment of shoulder disability in patients at one and six months postoperatively who had been operated on for neck dissection to determine the role played by simple physiotherapy exercises in their rehabilitation using the simple validated Shoulder Pain and Disability Index questionnaire [20].

The main result obtained was a significant reduction in pain, which made the patient very happy and motivated her to continue the treatment. After two sessions, there was a considerable improvement in the VAS score and an increase in the ROM [21].

## Conclusions

DN can be effective in patients undergoing neck dissection to improve ROM and reduce pain scores. DN is a semi-invasive method that can improve patient compliance with physiotherapy and rehabilitation. Further research is required to determine whether DN can be used alone or in conjunction with physiotherapy, and the study needs to be conducted with a larger sample size with randomization to prove or disprove the same.
